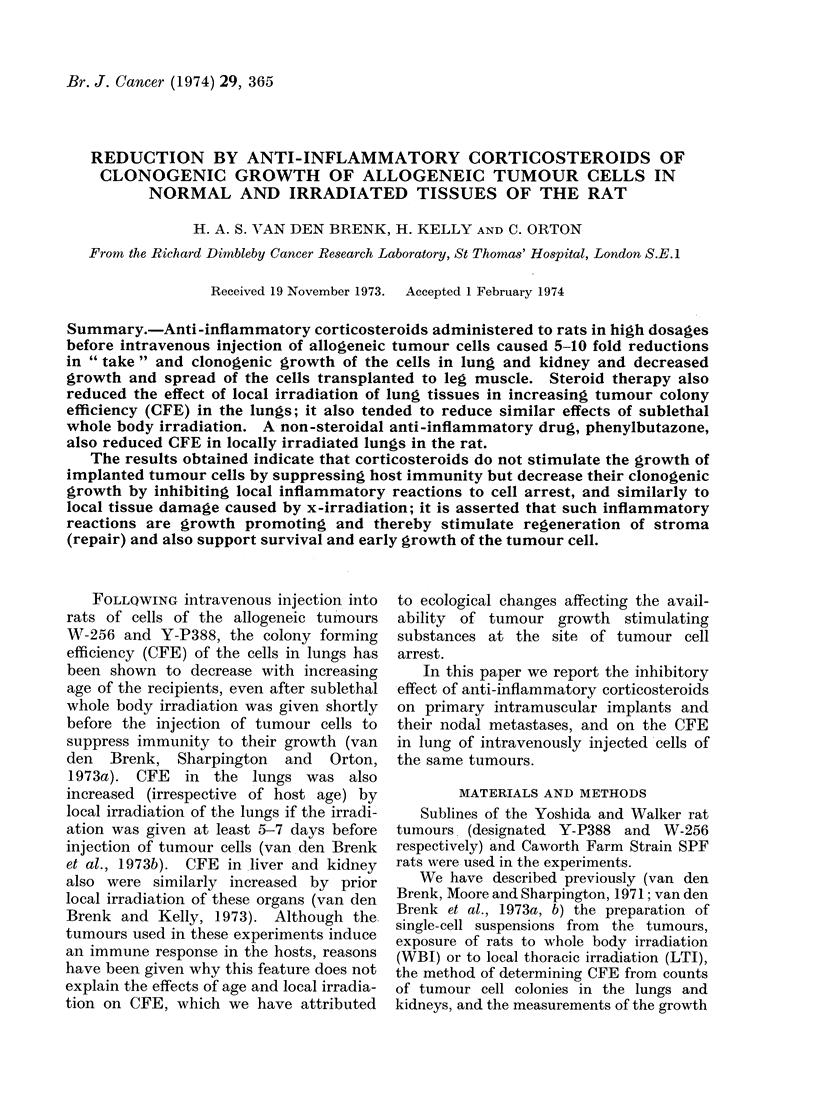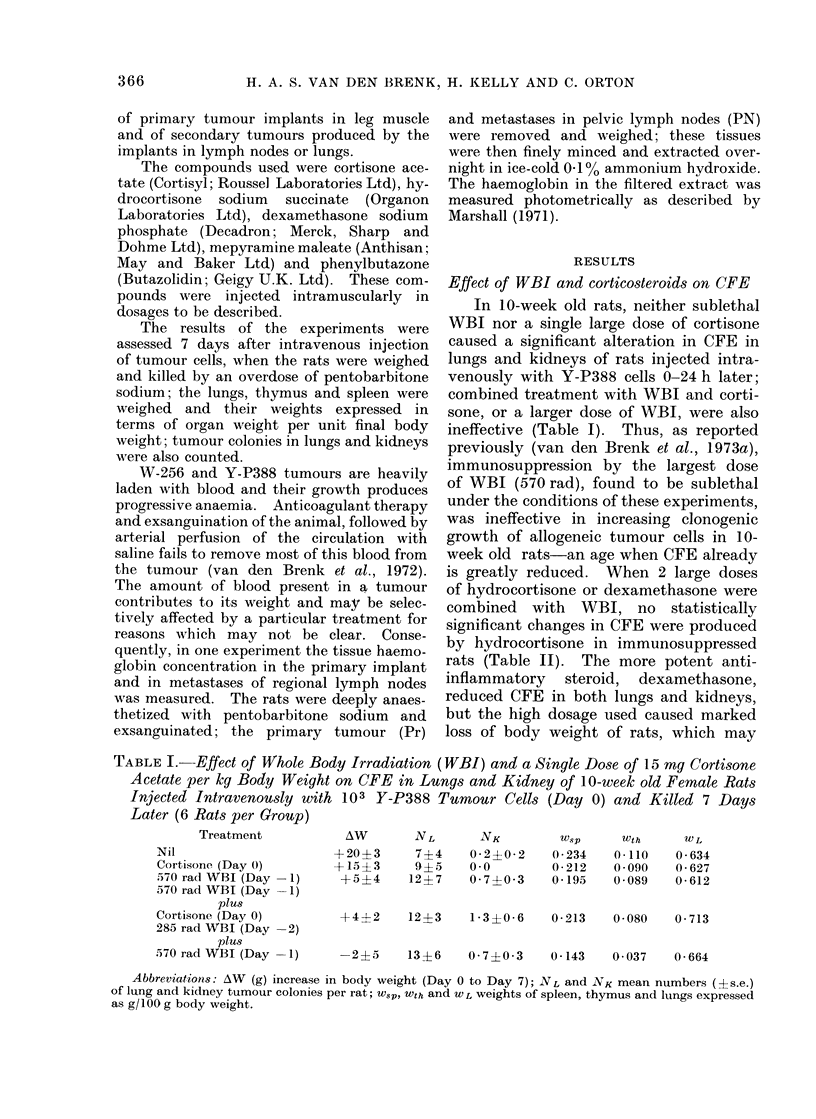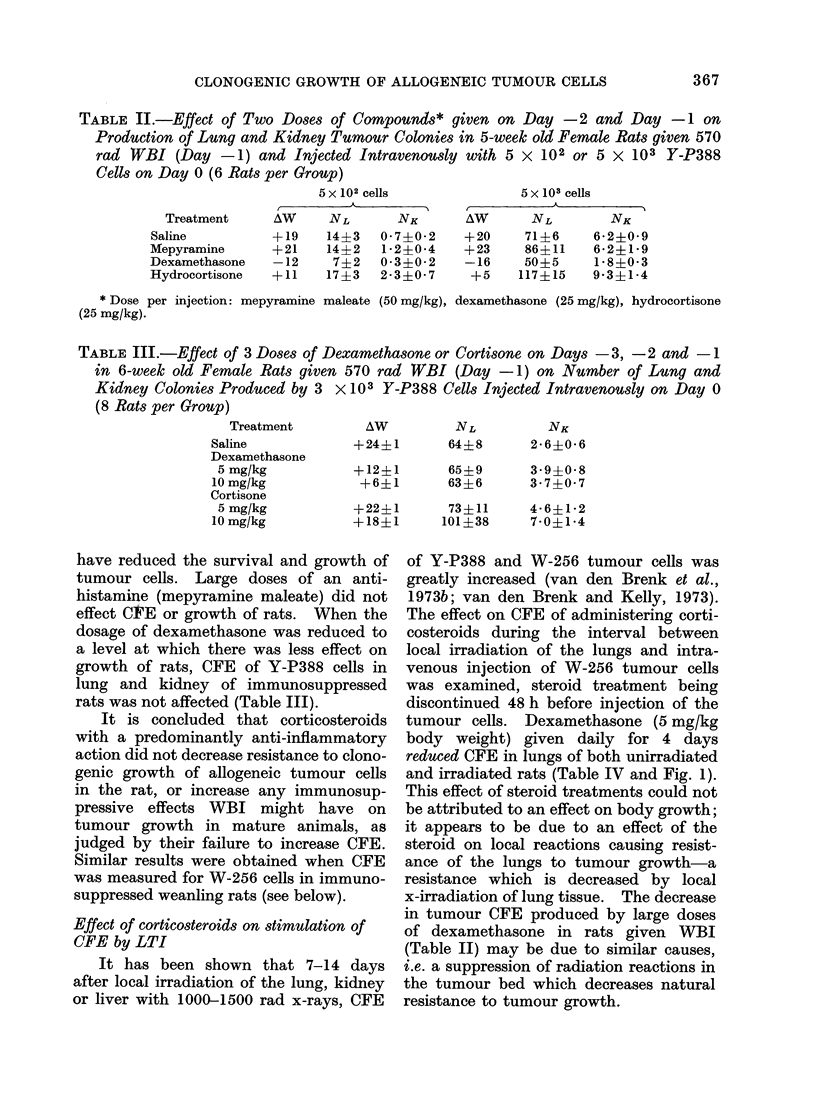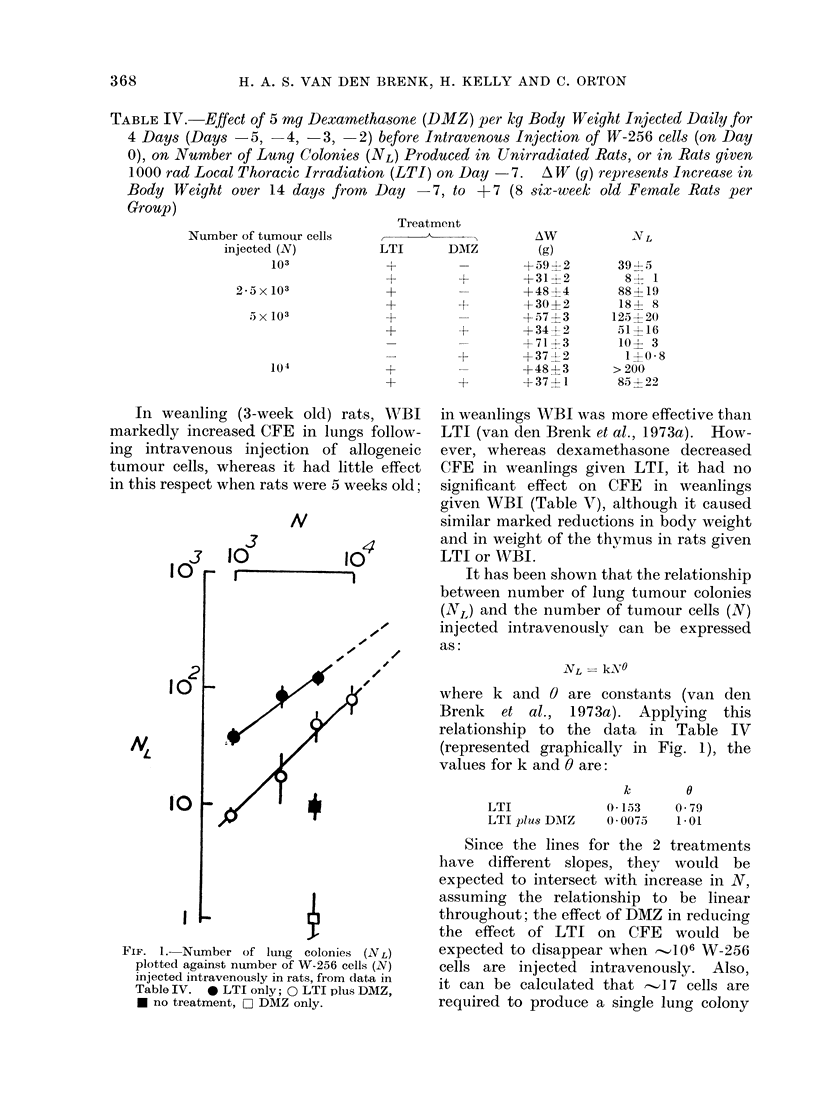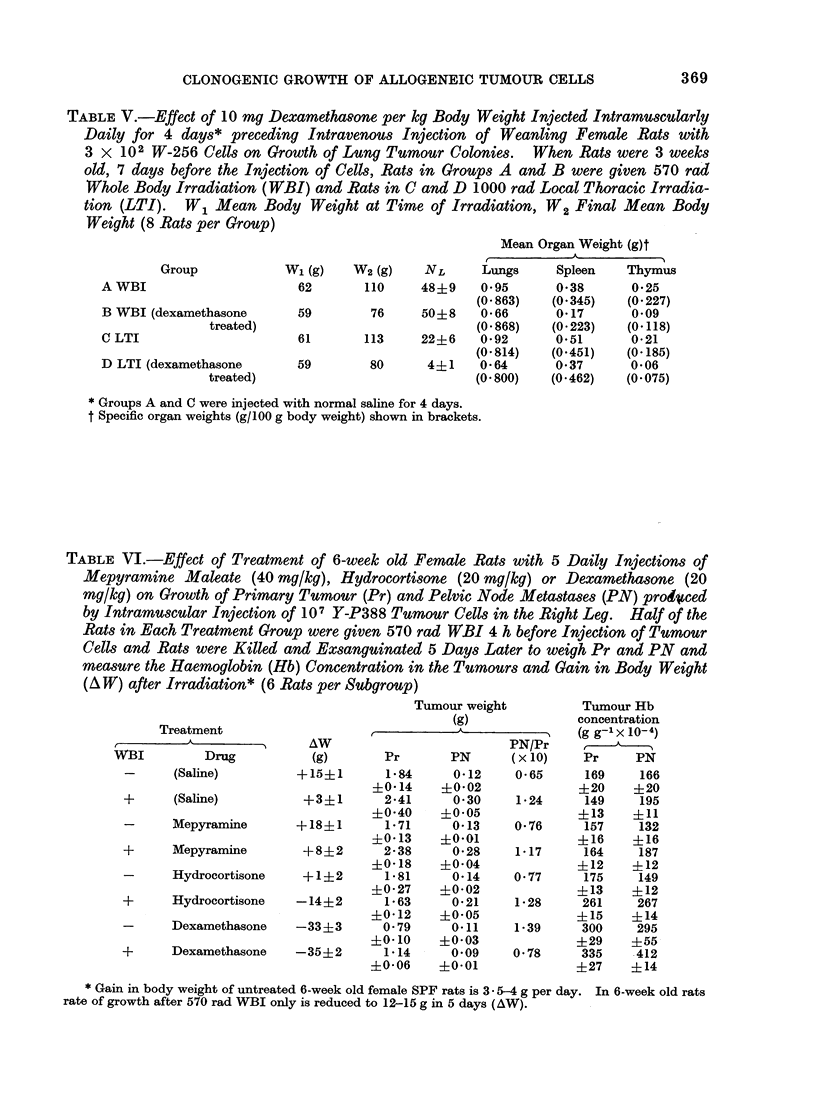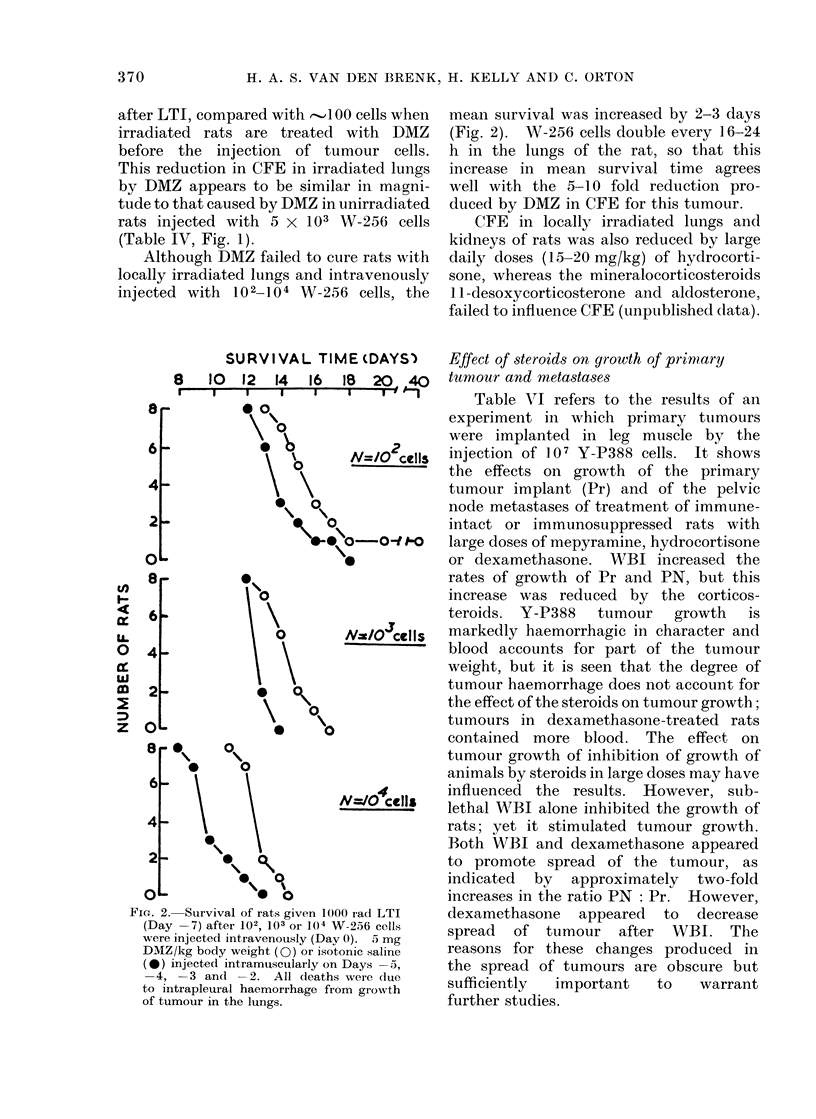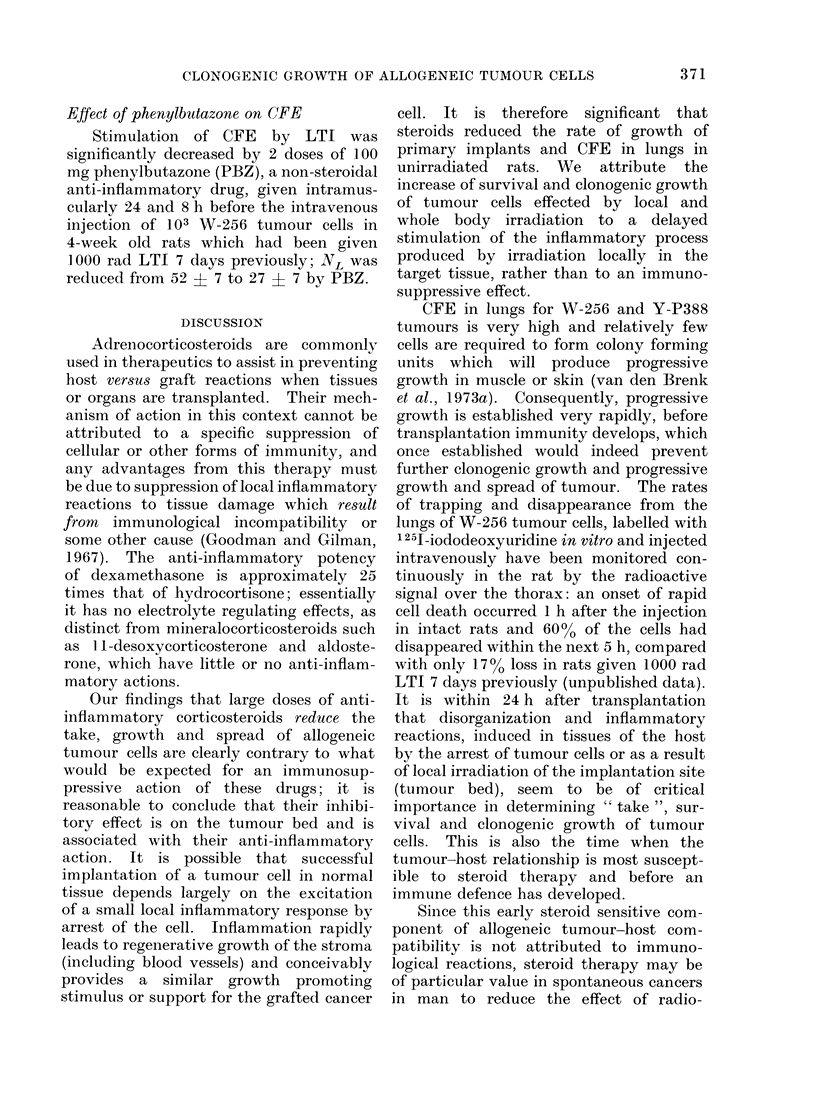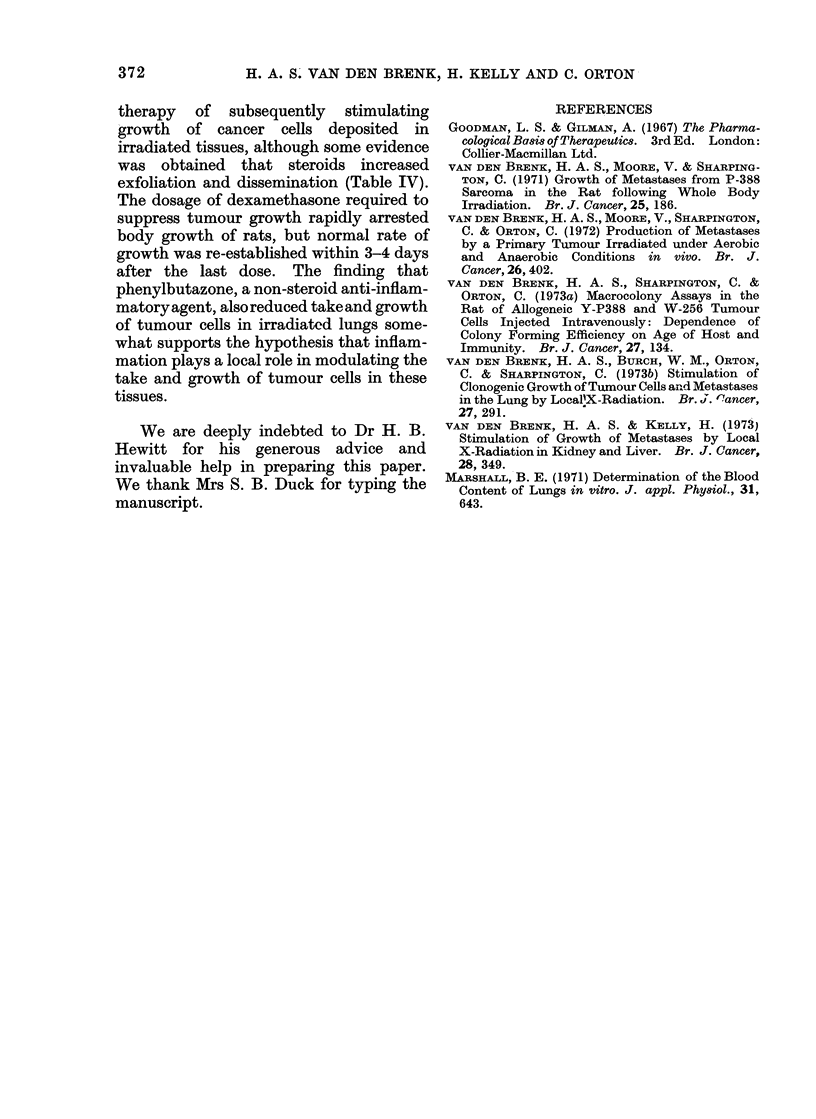# Reduction by Anti-Inflammatory Corticosteroids of Clonogenic Growth of Allogeneic Tumour Cells in Normal and Irradiated Tissues of the Rat

**DOI:** 10.1038/bjc.1974.84

**Published:** 1974-05

**Authors:** H. A. S. Van den Brenk, H. Kelly, C. Orton

## Abstract

Anti-inflammatory corticosteroids administered to rats in high dosages before intravenous injection of allogeneic tumour cells caused 5-10 fold reductions in “take” and clonogenic growth of the cells in lung and kidney and decreased growth and spread of the cells transplanted to leg muscle. Steroid therapy also reduced the effect of local irradiation of lung tissues in increasing tumour colony efficiency (CFE) in the lungs; it also tended to reduce similar effects of sublethal whole body irradiation. A non-steroidal anti-inflammatory drug, phenylbutazone, also reduced CFE in locally irradiated lungs in the rat.

The results obtained indicate that corticosteroids do not stimulate the growth of implanted tumour cells by suppressing host immunity but decrease their clonogenic growth by inhibiting local inflammatory reactions to cell arrest, and similarly to local tissue damage caused by x-irradiation; it is asserted that such inflammatory reactions are growth promoting and thereby stimulate regeneration of stroma (repair) and also support survival and early growth of the tumour cell.


*Br. J. Cancer* (1974) **29**, 365

### REDUCTION BY ANTI-INFLAMMATORY CORTICOSTEROIDS OF CLONOGENIC GROWTH OF ALLOGENEIC TUMOUR CELLS IN NORMAL AND IRRADIATED TISSUES OF THE RAT

H. A. S. VAN DEN BRENK, H. KELLY AND C. ORTON

*From the Richard Dimbleby Cancer Research Laboratory, St Thomas' Hospital, London S.E.*1

Received 19 November 1973. Accepted 1 February 1974

**Summary.—Anti-inflammatory corticosteroids administered to rats in high dosages before intravenous injection of allogeneic tumour cells caused 5–10 fold reductions in " take " and clonogenic growth of the cells in lung and kidney and decreased growth and spread of the cells transplanted to leg muscle. Steroid therapy also reduced the effect of local irradiation of lung tissues in increasing tumour colony efficiency (CFE) in the lungs; it also tended to reduce similar effects of sublethal whole body irradiation. A non-steroidal anti-inflammatory drug, phenylbutazone, also reduced CFE in locally irradiated lungs in the rat.**

**The results obtained indicate that corticosteroids do not stimulate the growth of implanted tumour cells by suppressing host immunity but decrease their clonogenic growth by inhibiting local inflammatory reactions to cell arrest, and similarly to local tissue damage caused by x-irradiation; it is asserted that such inflammatory reactions are growth promoting and thereby stimulate regeneration of stroma (repair) and also support survival and early growth of the tumour cell.**

FOLLOWING intravenous injection into rats of cells of the allogeneic tumours W-256 and Y-P388, the colony forming efficiency (CFE) of the cells in lungs has been shown to decrease with increasing age of the recipients, even after sublethal whole body irradiation was given shortly before the injection of tumour cells to suppress immunity to their growth (van den Brenk, Sharpington and Orton, 1973*a*). CFE in the lungs was also increased (irrespective of host age) by local irradiation of the lungs if the irradiation was given at least 5–7 days before injection of tumour cells (van den Brenk *et al.*, 1973*b*). CFE in liver and kidney also were similarly increased by prior local irradiation of these organs (van den Brenk and Kelly, 1973). Although the tumours used in these experiments induce an immune response in the hosts, reasons have been given why this feature does not explain the effects of age and local irradiation on CFE, which we have attributed to ecological changes affecting the availability of tumour growth stimulating substances at the site of tumour cell arrest.

In this paper we report the inhibitory effect of anti-inflammatory corticosteroids on primary intramuscular implants and their nodal metastases, and on the CFE in lung of intravenously injected cells of the same tumours.

### MATERIALS AND METHODS

Sublines of the Yoshida and Walker rat tumours (designated Y-P388 and W-256 respectively) and Caworth Farm Strain SPF rats were used in the experiments.

We have described previously (van den Brenk, Moore and Sharpington, 1971; van den Brenk *et al.*, 1973*a*, *b*) the preparation of single-cell suspensions from the tumours, exposure of rats to whole body irradiation (WBI) or to local thoracic irradiation (LTI), the method of determining CFE from counts of tumour cell colonies in the lungs and kidneys, and the measurements of the growth of primary tumour implants in leg muscle and of secondary tumours produced by the implants in lymph nodes or lungs.

The compounds used were cortisone acetate (Cortisyl; Roussel Laboratories Ltd), hydrocortisone sodium succinate (Organon Laboratories Ltd), dexamethasone sodium phosphate (Decadron; Merck, Sharp and Dohme Ltd), mepyramine maleate (Anthisan; May and Baker Ltd) and phenylbutazone (Butazolidin; Geigy U.K. Ltd). These compounds were injected intramuscularly in dosages to be described.

The results of the experiments were assessed 7 days after intravenous injection of tumour cells, when the rats were weighed and killed by an overdose of pentobarbitone sodium; the lungs, thymus and spleen were weighed and their weights expressed in terms of organ weight per unit final body weight; tumour colonies in lungs and kidneys were also counted.

W-256 and Y-P388 tumours are heavily laden with blood and their growth produces progressive anaemia. Anticoagulant therapy and exsanguination of the animal, followed by arterial perfusion of the circulation with saline fails to remove most of this blood from the tumour (van den Brenk *et al.*, 1972). The amount of blood present in a tumour contributes to its weight and may be selectively affected by a particular treatment for reasons which may not be clear. Consequently, in one experiment the tissue haemoglobin concentration in the primary implant and in metastases of regional lymph nodes was measured. The rats were deeply anaesthetized with pentobarbitone sodium and exsanguinated; the primary tumour (Pr) and metastases in pelvic lymph nodes (PN) were removed and weighed; these tissues were then finely minced and extracted overnight in ice-cold 0·1% ammonium hydroxide. The haemoglobin in the filtered extract was measured photometrically as described by Marshall (1971).

### RESULTS

### *Effect of WBI and corticosteroids on CFE*

In 10-week old rats, neither sublethal WBI nor a single large dose of cortisone caused a significant alteration in CFE in lungs and kidneys of rats injected intravenously with Y-P388 cells 0–24 h later; combined treatment with WBI and cortisone, or a larger dose of WBI, were also ineffective (Table I). Thus, as reported previously (van den Brenk *et al.*, 1973*a*), immunosuppression by the largest dose of WBI (570 rad), found to be sublethal under the conditions of these experiments, was ineffective in increasing clonogenic growth of allogeneic tumour cells in 10-week old rats—an age when CFE already is greatly reduced. When 2 large doses of hydrocortisone or dexamethasone were combined with WBI, no statistically significant changes in CFE were produced by hydrocortisone in immunosuppressed rats (Table II). The more potent anti-inflammatory steroid, dexamethasone, reduced CFE in both lungs and kidneys, but the high dosage used caused marked loss of body weight of rats, which may

TABLE I.—*Effect of Whole Body Irradiation* (*WBI*) *and a Single Dose of* 15 *mg Cortisone Acetate per kg Body Weight on CFE in Lungs and Kidney of* 10-*week old Female Rats Injected Intravenously with* $10^3$ *Y-P*388 *Tumour Cells* (*Day* 0) *and Killed* 7 *Days Later* (6 *Rats per Group*)

| Treatment | ΔW | $N_L$ | $N_K$ | $w_{sp}$ | $w_{th}$ | $w_L$ |
|---|---|---|---|---|---|---|
| Nil | +20±3 | 7±4 | 0·2±0·2 | 0·234 | 0·110 | 0·634 |
| Cortisone (Day 0) | +15±3 | 9±5 | 0·0 | 0·212 | 0·090 | 0·627 |
| 570 rad WBI (Day −1) | +5±4 | 12±7 | 0·7±0·3 | 0·195 | 0·089 | 0·612 |
| 570 rad WBI (Day −1) *plus* Cortisone (Day 0) | +4±2 | 12±3 | 1·3±0·6 | 0·213 | 0·080 | 0·713 |
| 285 rad WBI (Day −2) *plus* 570 rad WBI (Day −1) | −2±5 | 13±6 | 0·7±0·3 | 0·143 | 0·037 | 0·664 |

*Abbreviations:* ΔW (g) increase in body weight (Day 0 to Day 7); $N_L$ and $N_K$ mean numbers (±s.e.) of lung and kidney tumour colonies per rat; $w_{sp}$, $w_{th}$ and $w_L$ weights of spleen, thymus and lungs expressed as g/100 g body weight.

TABLE II.—*Effect of Two Doses of Compounds* given on Day —2 and Day —1 on Production of Lung and Kidney Tumour Colonies in 5-week old Female Rats given 570 rad WBI (Day —1) and Injected Intravenously with 5 × 10² or 5 × 10³ Y-P388 Cells on Day 0 (6 Rats per Group)*

| | $5 \times 10^2$ cells | | | $5 \times 10^3$ cells | | |
|---|---|---|---|---|---|---|
| Treatment | ΔW | $N_L$ | $N_K$ | ΔW | $N_L$ | $N_K$ |
| Saline | +19 | 14±3 | 0·7±0·2 | +20 | 71±6 | 6·2±0·9 |
| Mepyramine | +21 | 14±2 | 1·2±0·4 | +23 | 86±11 | 6·2±1·9 |
| Dexamethasone | −12 | 7±2 | 0·3±0·2 | −16 | 50±5 | 1·8±0·3 |
| Hydrocortisone | +11 | 17±3 | 2·3±0·7 | +5 | 117±15 | 9·3±1·4 |

* Dose per injection: mepyramine maleate (50 mg/kg), dexamethasone (25 mg/kg), hydrocortisone (25 mg/kg).

TABLE III.—*Effect of 3 Doses of Dexamethasone or Cortisone on Days —3, —2 and —1 in 6-week old Female Rats given 570 rad WBI (Day —1) on Number of Lung and Kidney Colonies Produced by 3 ×10³ Y-P388 Cells Injected Intravenously on Day 0 (8 Rats per Group)*

| Treatment | ΔW | $N_L$ | $N_K$ |
|---|---|---|---|
| Saline | +24±1 | 64±8 | 2·6±0·6 |
| Dexamethasone | | | |
| 5 mg/kg | +12±1 | 65±9 | 3·9±0·8 |
| 10 mg/kg | +6±1 | 63±6 | 3·7±0·7 |
| Cortisone | | | |
| 5 mg/kg | +22±1 | 73±11 | 4·6±1·2 |
| 10 mg/kg | +18±1 | 101±38 | 7·0±1·4 |

have reduced the survival and growth of tumour cells. Large doses of an antihistamine (mepyramine maleate) did not effect CFE or growth of rats. When the dosage of dexamethasone was reduced to a level at which there was less effect on growth of rats, CFE of Y-P388 cells in lung and kidney of immunosuppressed rats was not affected (Table III).

It is concluded that corticosteroids with a predominantly anti-inflammatory action did not decrease resistance to clonogenic growth of allogeneic tumour cells in the rat, or increase any immunosuppressive effects WBI might have on tumour growth in mature animals, as judged by their failure to increase CFE. Similar results were obtained when CFE was measured for W-256 cells in immunosuppressed weanling rats (see below).

*Effect of corticosteroids on stimulation of CFE by LTI*

It has been shown that 7–14 days after local irradiation of the lung, kidney or liver with 1000–1500 rad x-rays, CFE of Y-P388 and W-256 tumour cells was greatly increased (van den Brenk *et al.*, 1973*b*; van den Brenk and Kelly, 1973). The effect on CFE of administering corticosteroids during the interval between local irradiation of the lungs and intravenous injection of W-256 tumour cells was examined, steroid treatment being discontinued 48 h before injection of the tumour cells. Dexamethasone (5 mg/kg body weight) given daily for 4 days *reduced* CFE in lungs of both unirradiated and irradiated rats (Table IV and Fig. 1). This effect of steroid treatments could not be attributed to an effect on body growth; it appears to be due to an effect of the steroid on local reactions causing resistance of the lungs to tumour growth—a resistance which is decreased by local x-irradiation of lung tissue. The decrease in tumour CFE produced by large doses of dexamethasone in rats given WBI (Table II) may be due to similar causes, *i.e.* a suppression of radiation reactions in the tumour bed which decreases natural resistance to tumour growth.

TABLE IV.—*Effect of 5 mg Dexamethasone (DMZ) per kg Body Weight Injected Daily for 4 Days (Days —5, —4, —3, —2) before Intravenous Injection of W-256 cells (on Day 0), on Number of Lung Colonies ($N_L$) Produced in Unirradiated Rats, or in Rats given 1000 rad Local Thoracic Irradiation (LTI) on Day —7. $\Delta W$ (g) represents Increase in Body Weight over 14 days from Day —7, to +7 (8 six-week old Female Rats per Group)*

| Number of tumour cells injected ($N$) | Treatment LTI | Treatment DMZ | ΔW (g) | $N_L$ |
|---|---|---|---|---|
| $10^3$ | + | — | +59±2 | 39±5 |
| | + | + | +31±2 | 8± 1 |
| $2 \cdot 5 \times 10^3$ | + | — | +48±4 | 88±19 |
| | + | + | +30±2 | 18± 8 |
| $5 \times 10^3$ | + | — | +57±3 | 125±20 |
| | + | + | +34±2 | 51±16 |
| | — | — | +71±3 | 10± 3 |
| | — | + | +37±2 | 1±0·8 |
| $10^4$ | + | — | +48±3 | >200 |
| | + | + | +37±1 | 85±22 |

In weanling (3-week old) rats, WBI markedly increased CFE in lungs following intravenous injection of allogeneic tumour cells, whereas it had little effect in this respect when rats were 5 weeks old; in weanlings WBI was more effective than LTI (van den Brenk *et al.*, 1973*a*). However, whereas dexamethasone decreased CFE in weanlings given LTI, it had no significant effect on CFE in weanlings given WBI (Table V), although it caused similar marked reductions in body weight and in weight of the thymus in rats given LTI or WBI.

FIF. 1.—Number of lung colonies ($N_L$) plotted against number of W-256 cells ($N$) injected intravenously in rats, from data in Table IV. ● LTI only; ○ LTI plus DMZ, ■ no treatment, □ DMZ only.

It has been shown that the relationship between number of lung tumour colonies ($N_L$) and the number of tumour cells ($N$) injected intravenously can be expressed as:

$$N_L = kN^{\theta}$$

where k and $\theta$ are constants (van den Brenk *et al.*, 1973*a*). Applying this relationship to the data in Table IV (represented graphically in Fig. 1), the values for k and $\theta$ are:

| | *k* | $\theta$ |
|---|---|---|
| LTI | 0·153 | 0·79 |
| LTI *plus* DMZ | 0·0075 | 1·01 |

Since the lines for the 2 treatments have different slopes, they would be expected to intersect with increase in $N$, assuming the relationship to be linear throughout; the effect of DMZ in reducing the effect of LTI on CFE would be expected to disappear when $\sim 10^6$ W-256 cells are injected intravenously. Also, it can be calculated that $\sim$17 cells are required to produce a single lung colony

TABLE V.—*Effect of* 10 *mg Dexamethasone per kg Body Weight Injected Intramuscularly Daily for* 4 *days* preceding Intravenous Injection of Weanling Female Rats with* 3 × $10^2$ *W*-256 *Cells on Growth of Lung Tumour Colonies. When Rats were* 3 *weeks old,* 7 *days before the Injection of Cells, Rats in Groups A and B were given* 570 *rad Whole Body Irradiation* (*WBI*) *and Rats in C and D* 1000 *rad Local Thoracic Irradiation* (*LTI*). $W_1$ *Mean Body Weight at Time of Irradiation,* $W_2$ *Final Mean Body Weight* (8 *Rats per Group*)

| Group | $W_1$ (g) | $W_2$ (g) | $N_L$ | Mean Organ Weight (g)† Lungs | Spleen | Thymus |
|---|---|---|---|---|---|---|
| A WBI | 62 | 110 | 48±9 | 0·95 (0·863) | 0·38 (0·345) | 0·25 (0·227) |
| B WBI (dexamethasone treated) | 59 | 76 | 50±8 | 0·66 (0·868) | 0·17 (0·223) | 0·09 (0·118) |
| C LTI | 61 | 113 | 22±6 | 0·92 (0·814) | 0·51 (0·451) | 0·21 (0·185) |
| D LTI (dexamethasone treated) | 59 | 80 | 4±1 | 0·64 (0·800) | 0·37 (0·462) | 0·06 (0·075) |

* Groups A and C were injected with normal saline for 4 days.
† Specific organ weights (g/100 g body weight) shown in brackets.

TABLE VI.—*Effect of Treatment of* 6-*week old Female Rats with* 5 *Daily Injections of Mepyramine Maleate* (40 *mg/kg*), *Hydrocortisone* (20 *mg/kg*) *or Dexamethasone* (20 *mg/kg*) *on Growth of Primary Tumour* (*Pr*) *and Pelvic Node Metastases* (*PN*) *produced by Intramuscular Injection of* $10^7$ *Y-P*388 *Tumour Cells in the Right Leg. Half of the Rats in Each Treatment Group were given* 570 *rad WBI* 4 *h before Injection of Tumour Cells and Rats were Killed and Exsanguinated* 5 *Days Later to weigh Pr and PN and measure the Haemoglobin* (*Hb*) *Concentration in the Tumours and Gain in Body Weight* (Δ*W*) *after Irradiation** (6 *Rats per Subgroup*)

| Treatment WBI | Drug | ΔW (g) | Tumour weight (g) Pr | PN | PN/Pr (×10) | Tumour Hb concentration (g g⁻¹×10⁻⁴) Pr | PN |
|---|---|---|---|---|---|---|---|
| − | (Saline) | +15±1 | 1·84 ±0·14 | 0·12 ±0·02 | 0·65 | 169 ±20 | 166 ±20 |
| + | (Saline) | +3±1 | 2·41 ±0·40 | 0·30 ±0·05 | 1·24 | 149 ±13 | 195 ±11 |
| − | Mepyramine | +18±1 | 1·71 ±0·13 | 0·13 ±0·01 | 0·76 | 157 ±16 | 132 ±16 |
| + | Mepyramine | +8±2 | 2·38 ±0·18 | 0·28 ±0·04 | 1·17 | 164 ±12 | 187 ±12 |
| − | Hydrocortisone | +1±2 | 1·81 ±0·27 | 0·14 ±0·02 | 0·77 | 175 ±13 | 149 ±12 |
| + | Hydrocortisone | −14±2 | 1·63 ±0·12 | 0·21 ±0·05 | 1·28 | 261 ±15 | 267 ±14 |
| − | Dexamethasone | −33±3 | 0·79 ±0·10 | 0·11 ±0·03 | 1·39 | 300 ±29 | 295 ±55 |
| + | Dexamethasone | −35±2 | 1·14 ±0·06 | 0·09 ±0·01 | 0·78 | 335 ±27 | 412 ±14 |

* Gain in body weight of untreated 6-week old female SPF rats is 3·5–4 g per day. In 6-week old rats rate of growth after 570 rad WBI only is reduced to 12–15 g in 5 days (ΔW).

after LTI, compared with ~100 cells when irradiated rats are treated with DMZ before the injection of tumour cells. This reduction in CFE in irradiated lungs by DMZ appears to be similar in magnitude to that caused by DMZ in unirradiated rats injected with $5 \times 10^3$ W-256 cells (Table IV, Fig. 1).

Although DMZ failed to cure rats with locally irradiated lungs and intravenously injected with $10^2$–$10^4$ W-256 cells, the mean survival was increased by 2–3 days (Fig. 2). W-256 cells double every 16–24 h in the lungs of the rat, so that this increase in mean survival time agrees well with the 5–10 fold reduction produced by DMZ in CFE for this tumour.

CFE in locally irradiated lungs and kidneys of rats was also reduced by large daily doses (15–20 mg/kg) of hydrocortisone, whereas the mineralocorticosteroids 11-desoxycorticosterone and aldosterone, failed to influence CFE (unpublished data).

Fig. 2.—Survival of rats given 1000 rad LTI (Day −7) after $10^2$, $10^3$ or $10^4$ W-256 cells were injected intravenously (Day 0). 5 mg DMZ/kg body weight (○) or isotonic saline (●) injected intramuscularly on Days −5, −4, −3 and −2. All deaths were due to intrapleural haemorrhage from growth of tumour in the lungs.

*Effect of steroids on growth of primary tumour and metastases*

Table VI refers to the results of an experiment in which primary tumours were implanted in leg muscle by the injection of $10^7$ Y-P388 cells. It shows the effects on growth of the primary tumour implant (Pr) and of the pelvic node metastases of treatment of immune-intact or immunosuppressed rats with large doses of mepyramine, hydrocortisone or dexamethasone. WBI increased the rates of growth of Pr and PN, but this increase was reduced by the corticosteroids. Y-P388 tumour growth is markedly haemorrhagic in character and blood accounts for part of the tumour weight, but it is seen that the degree of tumour haemorrhage does not account for the effect of the steroids on tumour growth; tumours in dexamethasone-treated rats contained more blood. The effect on tumour growth of inhibition of growth of animals by steroids in large doses may have influenced the results. However, sublethal WBI alone inhibited the growth of rats; yet it stimulated tumour growth. Both WBI and dexamethasone appeared to promote spread of the tumour, as indicated by approximately two-fold increases in the ratio PN : Pr. However, dexamethasone appeared to decrease spread of tumour after WBI. The reasons for these changes produced in the spread of tumours are obscure but sufficiently important to warrant further studies.

*Effect of phenylbutazone on CFE*

Stimulation of CFE by LTI was significantly decreased by 2 doses of 100 mg phenylbutazone (PBZ), a non-steroidal anti-inflammatory drug, given intramuscularly 24 and 8 h before the intravenous injection of $10^3$ W-256 tumour cells in 4-week old rats which had been given 1000 rad LTI 7 days previously; $N_L$ was reduced from 52 $\pm$ 7 to 27 $\pm$ 7 by PBZ.

### DISCUSSION

Adrenocorticosteroids are commonly used in therapeutics to assist in preventing host *versus* graft reactions when tissues or organs are transplanted. Their mechanism of action in this context cannot be attributed to a specific suppression of cellular or other forms of immunity, and any advantages from this therapy must be due to suppression of local inflammatory reactions to tissue damage which *result from* immunological incompatibility or some other cause (Goodman and Gilman, 1967). The anti-inflammatory potency of dexamethasone is approximately 25 times that of hydrocortisone; essentially it has no electrolyte regulating effects, as distinct from mineralocorticosteroids such as 11-desoxycorticosterone and aldosterone, which have little or no anti-inflammatory actions.

Our findings that large doses of anti-inflammatory corticosteroids *reduce* the take, growth and spread of allogeneic tumour cells are clearly contrary to what would be expected for an immunosuppressive action of these drugs; it is reasonable to conclude that their inhibitory effect is on the tumour bed and is associated with their anti-inflammatory action. It is possible that successful implantation of a tumour cell in normal tissue depends largely on the excitation of a small local inflammatory response by arrest of the cell. Inflammation rapidly leads to regenerative growth of the stroma (including blood vessels) and conceivably provides a similar growth promoting stimulus or support for the grafted cancer cell. It is therefore significant that steroids reduced the rate of growth of primary implants and CFE in lungs in unirradiated rats. We attribute the increase of survival and clonogenic growth of tumour cells effected by local and whole body irradiation to a delayed stimulation of the inflammatory process produced by irradiation locally in the target tissue, rather than to an immunosuppressive effect.

CFE in lungs for W-256 and Y-P388 tumours is very high and relatively few cells are required to form colony forming units which will produce progressive growth in muscle or skin (van den Brenk *et al.*, 1973*a*). Consequently, progressive growth is established very rapidly, before transplantation immunity develops, which once established would indeed prevent further clonogenic growth and progressive growth and spread of tumour. The rates of trapping and disappearance from the lungs of W-256 tumour cells, labelled with $^{125}$I-iododeoxyuridine *in vitro* and injected intravenously have been monitored continuously in the rat by the radioactive signal over the thorax: an onset of rapid cell death occurred 1 h after the injection in intact rats and 60% of the cells had disappeared within the next 5 h, compared with only 17% loss in rats given 1000 rad LTI 7 days previously (unpublished data). It is within 24 h after transplantation that disorganization and inflammatory reactions, induced in tissues of the host by the arrest of tumour cells or as a result of local irradiation of the implantation site (tumour bed), seem to be of critical importance in determining " take ", survival and clonogenic growth of tumour cells. This is also the time when the tumour–host relationship is most susceptible to steroid therapy and before an immune defence has developed.

Since this early steroid sensitive component of allogeneic tumour–host compatibility is not attributed to immunological reactions, steroid therapy may be of particular value in spontaneous cancers in man to reduce the effect of radiotherapy of subsequently stimulating growth of cancer cells deposited in irradiated tissues, although some evidence was obtained that steroids increased exfoliation and dissemination (Table IV). The dosage of dexamethasone required to suppress tumour growth rapidly arrested body growth of rats, but normal rate of growth was re-established within 3–4 days after the last dose. The finding that phenylbutazone, a non-steroid anti-inflammatory agent, also reduced take and growth of tumour cells in irradiated lungs somewhat supports the hypothesis that inflammation plays a local role in modulating the take and growth of tumour cells in these tissues.

We are deeply indebted to Dr H. B. Hewitt for his generous advice and invaluable help in preparing this paper. We thank Mrs S. B. Duck for typing the manuscript.

### REFERENCES

GOODMAN, L. S. & GILMAN, A. (1967) *The Pharmacological Basis of Therapeutics*. 3rd Ed. London: Collier-Macmillan Ltd.

VAN DEN BRENK, H. A. S., MOORE, V. & SHARPINGTON, C. (1971) Growth of Metastases from P-388 Sarcoma in the Rat following Whole Body Irradiation. *Br. J. Cancer*, **25**, 186.

VAN DEN BRENK, H. A. S., MOORE, V., SHARPINGTON, C. & ORTON, C. (1972) Production of Metastases by a Primary Tumour Irradiated under Aerobic and Anaerobic Conditions *in vivo*. *Br. J. Cancer*, **26**, 402.

VAN DEN BRENK, H. A. S., SHARPINGTON, C. & ORTON, C. (1973*a*) Macrocolony Assays in the Rat of Allogeneic Y-P388 and W-256 Tumour Cells Injected Intravenously: Dependence of Colony Forming Efficiency on Age of Host and Immunity. *Br. J. Cancer*, **27**, 134.

VAN DEN BRENK, H. A. S., BURCH, W. M., ORTON, C. & SHARPINGTON, C. (1973*b*) Stimulation of Clonogenic Growth of Tumour Cells and Metastases in the Lung by Local X-Radiation. *Br. J. Cancer*, **27**, 291.

VAN DEN BRENK, H. A. S. & KELLY, H. (1973) Stimulation of Growth of Metastases by Local X-Radiation in Kidney and Liver. *Br. J. Cancer*, **28**, 349.

MARSHALL, B. E. (1971) Determination of the Blood Content of Lungs *in vitro*. *J. appl. Physiol.*, **31**, 643.